# Multiscale Autoregressive Identification of Neuroelectrophysiological Systems

**DOI:** 10.1155/2012/580795

**Published:** 2012-02-15

**Authors:** Timothy P. Gilmour, Thyagarajan Subramanian, Constantino Lagoa, W. Kenneth Jenkins

**Affiliations:** ^1^Electrical Engineering Department, Pennsylvania State University, University Park, PA 16802, USA; ^2^Neurology Department, Penn State Hershey Medical Center, 500 University Drive, Hershey, PA 17033, USA

## Abstract

Electrical signals between connected neural nuclei are difficult to model because of the complexity and high number of paths within the brain. Simple parametric models are therefore often used. A multiscale version of the autoregressive with exogenous input (MS-ARX) model has recently been developed which allows selection of the optimal amount of filtering and decimation depending on the signal-to-noise ratio and degree of predictability. In this paper, we apply the MS-ARX model to cortical electroencephalograms and subthalamic local field potentials simultaneously recorded from anesthetized rodent brains. We demonstrate that the MS-ARX model produces better predictions than traditional ARX modeling. We also adapt the MS-ARX results to show differences in internuclei predictability between normal rats and rats with 6OHDA-induced parkinsonism, indicating that this method may have broad applicability to other neuroelectrophysiological studies.

## 1. Introduction

VARIOUS types of methods have been used to assess the degree of similarity or shared information between two signals. The methods used depend on the type of the presumptive system which processes the one “input” signal into the other “output” signal. Two basic classifications of systems are whether they are memoryless or not, and whether they are linear or not.

Common linear memoryless methods include the cross-correlation in the time domain or coherence in the frequency domain. Common linear models with memory include autoregressive models with exogenous input (ARX), autoregressive moving average (ARMA), Box-Jenkins, Output-Error, and linear state-space models.

Higher-order nonlinear methods with memory are also sometimes used, such as polyspectral models, nonlinear ARX models, neural networks, Hammerstein-Wiener models, and Volterra models, but these are more difficult to train.

Other statistical evaluations of the similarity focus on the transfer of information between two signals, rather than explicit modeling and prediction. Examples of these analyses include Granger causality analysis, time-delay mutual information, and transfer entropy. The information theoretic analyses can measure nonlinear as well as linear effects.

Complex systems such as the brain are difficult to analyze because of the huge number of individual neuronal/synaptic paths between nuclei, the nonlinear nature of neuronal connections, and the operation at multiple time scales.

One approach is to use simple low-order linear models to approximate the transfer function relationship, such as autoregressive with exogenous (ARX) models. The advantage of using linear ARX models is that there is no need to estimate nonlinearity parameters, and less training data is required. However, the performance of such models depends crucially on the model order, scale, and prefiltering. To mitigate this dependency, a multiscale version of the autoregressive with exogenous input (MS-ARX) model has recently been developed by Nounou and colleagues [1]. The MS-ARX model allows automatic selection of the optimal scale for the ARX prediction.

In this paper, we adapt and apply the MS-ARX model to evaluate the degree of information transfer between cortical electroencephalogram (EEG) and subthalamic nucleus (STN) local field potential (LFP) signals. In a rat model of Parkinson's disease, the multiscale ARX approach showed significant differences in connectivity compared to normal.

## 2. Methods

### 2.1. Autoregressive System Identification

 The ARX model is a common method to represent output signals from an unknown system by using a linear combination of past output signal values and past input values. We will be following the notation of Nounou and colleagues in our model description. The equation for the ARX model is

(1) $$y\left(k+1\right)=\overset{p}{\underset{i=0}{\sum }}\alpha_{i}y\left(k-i\right)+\overset{q}{\underset{m=0}{\sum }}\beta_{m}u\left(k-m\right),$$

where *y* is the output, *u* is the input, *α*_*i*_ and *β*_*m*_ are the estimated system coefficients, and *p* and *q* are the maximum orders of the autoregressive and input filters, respectively. Equation (1) may be written in matrix form as

(2) Y=Xθ,

where

(3) $$\begin{array}{cc} & Y=\left[\begin{array}{c} y\left(n\right)\\ y\left(n-1\right)\\ y\left(n-2\right)\\ \vdots \end{array}\right],\;\;\theta ={\left[\alpha_{1}\cdots \alpha_{p}\;\beta_{1}\cdots \beta_{q}\right]}^{T},\\ X & =\left[\begin{array}{cccccc} y\left(n-1\right) & \cdots & y\left(n-p\right) & u\left(n-1\right) & \cdots & u\left(n-q\right)\\ y\left(n-2\right) & \cdots & y\left(n-p-1\right) & u\left(n-2\right) & \cdots & u\left(n-q-1\right)\\ y\left(n-3\right) & \cdots & y\left(n-p-2\right) & u\left(n-3\right) & \cdots & u\left(n-q-2\right)\\ \vdots & & \vdots & \vdots & & \vdots \end{array}\right]. \end{array}$$

The weight parameters *α*_*i*_ and *β*_*m*_ may be solved using least squares:

(4) $$\theta_{LS}={\left(X^{T}X\right)}^{-1}X^{T}Y.$$

The maximum filter lengths *p* and *q* may be estimated by minimizing some criterion such as the Akaike information criterion (AIC):

(5) AIC=r−2ln⁡(L),

where *r* is the number of model parameters, and *L* is the likelihood function quantifying the model goodness of fit.

#### 2.1.1. Wavelet Decomposition

Signals may be decomposed into a multiscale time-frequency representation by projecting the signal onto an orthonormal set of basic functions. These functions correspond to a particular scale and translation of a prototype scaling function *ϕ*_*jk*_(*t*) and wavelet function *ψ*_*jk*_(*t*), given by

(6) $$\begin{array}{c} \phi_{jk}\left(t\right)=\sqrt{2^{-j}}\phi \left(2^{-j}t-k\right),\\ \psi_{jk}\left(t\right)=\sqrt{2^{-j}}\psi \left(2^{-j}t-k\right). \end{array}$$

For the Haar wavelet used in this paper,

(7) $$\begin{array}{c} \phi \left(t\right)=\{\begin{array}{cc} 1, & 0\leq t\leq 1,\\ 0, & \left|t\right|>1, \end{array}\\ \psi \left(t\right)=\{\begin{array}{cc} 1, & 0\leq t<0.5,\\ -1, & 0.5\leq t\leq 1\\ 0, & \left|t\right|>1. \end{array} \end{array}$$

#### 2.1.2. Multiscale ARX Modeling

**Figure 1:**
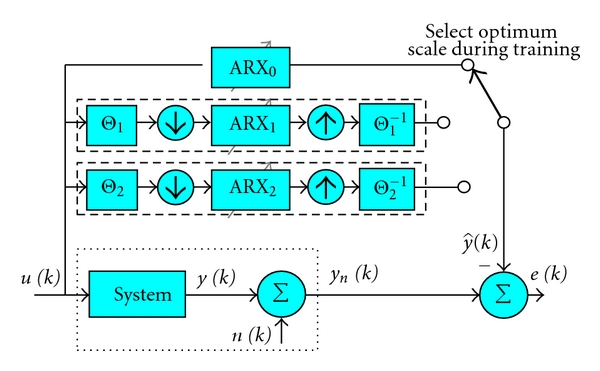
Block diagram of MS-ARX system-identification configuration. In general, the system (dotted box) is unknown and so the true output *y*(*k*) and measurement noise *n*(*k*) are unknown and only *y*_*n*_(*k*) is measurable. The multirate equivalence theorem stated by Nounou et al. allows precomputation of the scaled wavelet ARX prediction blocks (dashed boxes), reducing computational complexity.

We applied the multiscale ARX approach presented by Nounou and colleagues. Briefly, the input (EEG) and output (LFP) data were first split in half into a training and a validation set. Second, both sets were decomposed using Haar wavelets into multiple scaled approximations in addition to the original undecimated scale. Third, at each scale an ARX model was trained using the model structure selected by an AIC minimization. Fourth, the computed ARX model from each scale was converted to the original sampling rate using the following theorem proved by Nounou et al.: an ARX transfer function *Y*_*j*_(*z*)/*U*_*j*_(*z*) = *G*(*z*) at scale *j* is equivalent to the transfer function *Y*_0_(*z*)/*U*_0_(*z*) = *G*(*z* ^2*j*^) at scale 0, the original undecimated scale. Fifth, the optimal scale ARX model was selected as the one which provided the smallest mean-square error (MSE) on the validation set (Figure 1). The MSE is defined as

(8) $$\text{MSE}=\frac{1}{n}\overset{n}{\underset{k}{\sum }}{\left[\hat{y}(k)-y_{n}(k)\right]}^{2}.$$

#### 2.1.3. Neural Data Collection

The motor cortex has been shown to project into the subthalamic nucleus (STN), thus implying a system of unknown electrical parameters with the EEG as the input and the STN local field potential (LFP) as the output [2]. Furthermore, studies in preclinical models of Parkinson's disease have shown increased correlation between neighboring neurons [3] and increased coherence in the 15–30 Hz band between basal ganglia nuclei [4, 5].

To examine the connection strength between these disparate brain areas using MS-ARX, we simultaneously recorded voltage data from the motor cortex EEG and STN LFP of anesthetized normal rats and hemiparkinsonian (HP) rats. Parkinsonism was induced by the vendor (Charles River) by 6-hydroxydopamine injection (12 *μ*g in 4 *μ*L, injected into coordinates AP −1.5 mm, ML +1.8, DV −7.5 from dura at 0.67 *μ*L/minute, cf. [6, 7]) and was verified by apomorphine-induced rotation testing and histological verification as detailed elsewhere [7, 8]. Briefly, rats were injected with apomorphine HCl (0.2 mg/kg, subcutaneously) at 3 and 5 weeks after 6-OHDA exposure and the number of contralateral turns over 35 minutes were counted with an automated rotameter. Rats averaging more than 7 turns per minute were included in the HP group. After recording and euthanasia, the brain was frozen, sectioned coronally, stained for tyrosine hydroxylase to confirm 95% unilateral lesioning of the substantia nigra pars compacta, and stained with cresyl violet to confirm electrode localization. All procedures were approved by the Pennsylvania State University Institutional Animal Care and Use Committee.

For EEG recording, animals were deeply anesthetized with urethane, with nominal initial dose 1.3 g/kg (i.p.) and additional doses given as needed to maintain surgical anesthesia. Stainless steel screws were implanted above bregma and above motor cortex (AP +3.7 ± 1.0 mm, ML +2.5), and the EEG signal recorded as the potential difference between these screws was subsequently amplified and filtered between 0.1 Hz and 500 Hz (3500, A-M Systems) and digitized at an initial rate of 1000 samples per second. An occipital screw was used as the reference electrode. The LFP signal was taken from the tip of the tungsten microelectrode (1-2 MΩ, FHC Inc), filtered between 5 Hz–500 Hz, amplified, and digitized. Offline, both signals were filtered between 0.1 Hz and 100 Hz and downsampled to 200 samples per second.

Recordings were taken from 9 normal rats (37 distinct STN sites) and 8 HP rats (26 distinct STN sites). Electrode tracts were histologically confirmed.

We used a maximum of 260 taps in our AIC structure selection step. This maximum was empirically selected based on the observation that the peak frequencies in our data were usually between 0.8–1.3 Hz or higher, thus allowing at least one cycle period within the ARX filter length at scale zero. The maximum wavelet decomposition level was 4. Only recordings with robust slow-wave activity were included in the analysis [9]. 

## 3. Results

Table 1 shows the MSEs at different scales. The undecimated scale was the optimal scale for approximately half of the recordings. The other recordings saw better ARX prediction performance at higher wavelet scales (more heavily filtered wavelet approximations).

**Figure 2:**
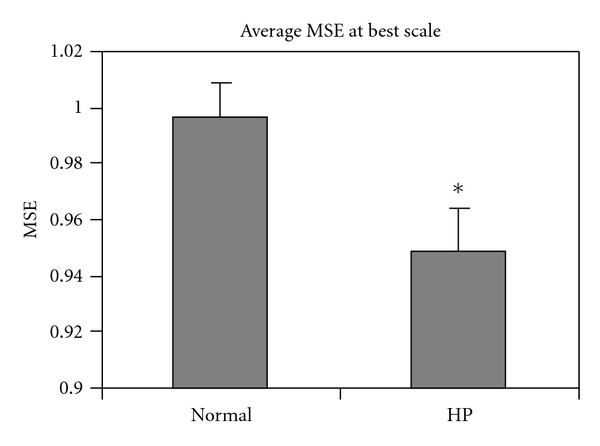
Mean MSE across all recordings at their optimal scale (*denotes *P* < 0.05 rank-sum test between Normal and HP groups).

**Figure 3:**
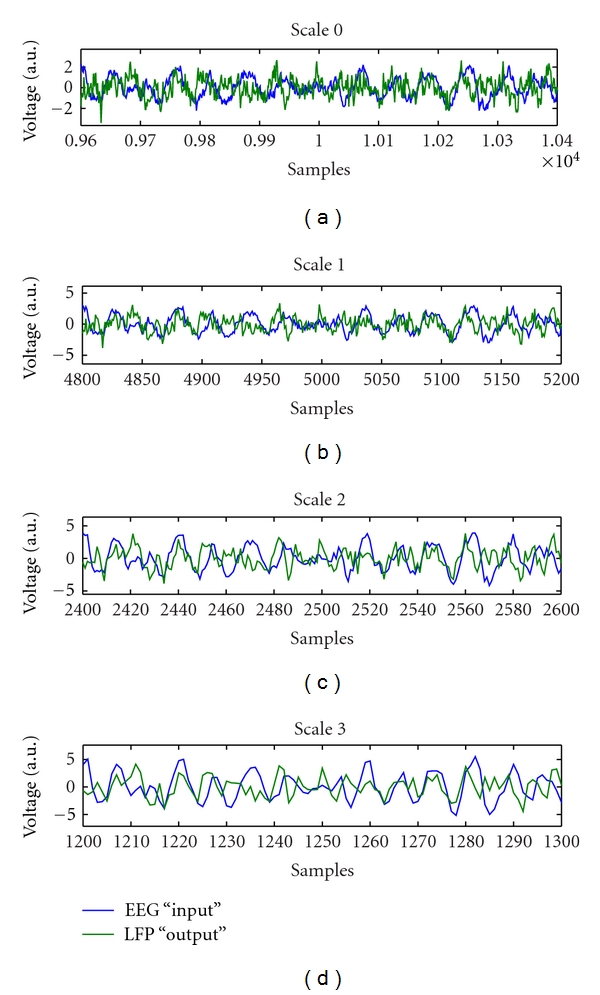
Sample EEG and LFP waveforms showing the original scale and three successive levels of scaled wavelet decimations.

**Figure 4:**
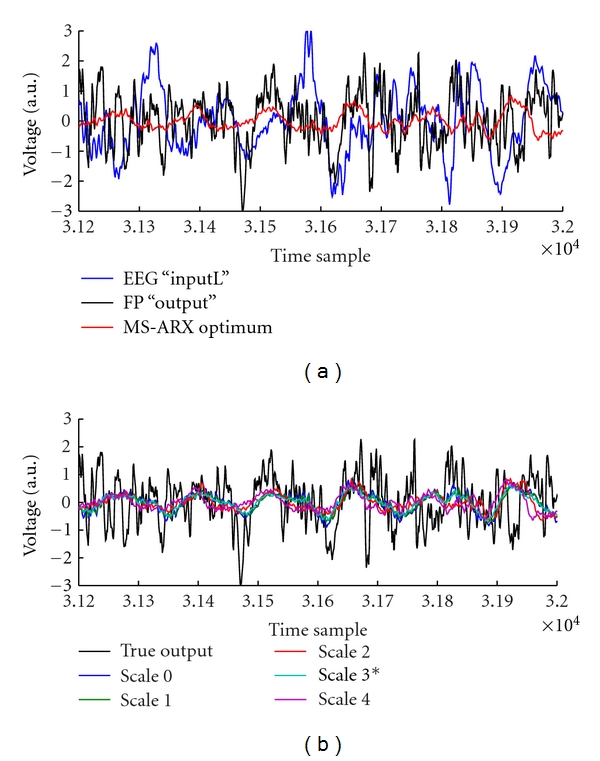
(a) Overlaid sample EEG input, LFP output, and the MS-ARX optimal scale prediction (scale 3). (b) Overlaid LFP output and predictions from all scales.

The mean MSE at the optimum scale was significantly lower in the HP group compared to the normal group (Figure 2, *P* < 0.05, rank-sum test). Also, the ratio (computed for each individual recording) of the mean of the absolute value of the best-scale AR coefficients (*α*_*i*_) to the mean of the absolute value of the best-scale exogenous input coefficients (*β*_*m*_) was significantly lower in the HP group (Table 2, *P* < 0.05, rank-sum test). Figures 3 and 4 show samples of the wavelet decimation and MS-ARX predictions, illustrating the differences between scales. 

## 4. Discussion

The MS-ARX technique showed improved prediction accuracy compared to the traditional ARX approach (which uses scale 0 only). This makes sense because the MS-ARX approach uses cross-validation to automatically select the best tradeoff between smoothing and preservation of signal details.

The results seen of decreased MSE and increased proportion of exogenous input weights also indicate that linear prediction of the STN LFP based on the cortical EEG is more accurate in the HP condition and is based more on cortical input. This indicates a greater amount of similarity between the population-based cortical and STN electrical activity in the HP case. Future studies should investigate whether this increased predictability is correlated to parkinsonian pathophysiology or symptomatic severity. Brown and colleagues have shown that there is increased coherence between the cortical EEG and STN LFP in the 15–30 Hz “beta” band in PD [4, 5, 10, 11], and also that STN LFP beta activity is correlated to clinical symptoms [12–14], suggesting that the linear predictability in those scales may also be correlated to clinical symptoms. However, unlike the coherence, the MS-ARX prediction measure we describe can also measure nonperiodic content similarity between the cortical EEG and STN LFP. Thus it may provide an additional useful tool to investigate nonperiodic corticosubthalamic interactions. The recent optogenetic study by Deisseroth and colleagues showed that high-frequency stimulation of the motor cortex achieved similar symptomatic amelioration as STN stimulation, presumably affecting the STN through the hyperdirect pathway [2, 15]. However, this effect was only seen in high-frequency stimulation, suggesting that the temporal scale of synchronization may be important. The multiscale predictability analysis presented here can aid in analyzing these scale-differential effects.

In conclusion, the MS-ARX method is well-adapted to the high-level analysis of neural signals from different brain nuclei at multiple scales.

## Figures and Tables

**Table 1:** Mean MSE at different scales across all recordings. Numbers in parentheses are the percent of neuronal recordings which selected that particular scale as optimum.

Scale	MSE (mean ± SEM)	
	Normal	HP
*j* = 0	1.005 ± 0.015 (51%)	0.953 ± 0.015 (56%)
*j* = 1	1.008 ± 0.014 (11%)	0.958 ± 0.014 (24%)
*j* = 2	1.012 ± 0.014 (3%)	0.968 ± 0.014 (4%)
*j* = 3	1.013 ± 0.012 (8%)	0.973 ± 0.012 (8%)
*j* = 4	1.019 ± 0.010 (27%)	1.000 ± 0.010 (8%)

**Table 2:** Parameter summary for normal and hemiparkinsonian recordings. Each row shows the mean (± SEM) absolute value of the parameter at the optimum scale for each recording (*denotes *P* < 0.05 rank-sum test between Normal and HP groups).

Mean of parameter	Normal	HP
AR coeffs.	0.21 ± 0.024	0.19 ± 0.025
Exogenous input (X) coeffs.	0.083 ± 0.016	0.15 ± 0.033
Ratio of AR to X coeffs.	6.4 ± 0.87	4.9 ± 1.26*
Number of nonzero AR coeffs.	157.3 ± 18.5	177.8 ± 19.3
Number of nonzero X coeffs.	156.3 ± 18.5	176.9 ± 19.3
Best scale	1.49 ± 0.29	1.00 ± 0.27
